# Synthesis of the Oligosaccharides Related to Branching Sites of Fucosylated Chondroitin Sulfates from Sea Cucumbers

**DOI:** 10.3390/md13020770

**Published:** 2015-02-02

**Authors:** Nadezhda E. Ustyuzhanina, Polina A. Fomitskaya, Alexey G. Gerbst, Andrey S. Dmitrenok, Nikolay E. Nifantiev

**Affiliations:** 1 N.D. Zelinsky Institute of Organic Chemistry, Russian Academy of Sciences, Leninsky prospect 47, 119991 Moscow B-334, Russia; E-Mails: alger@ioc.ac.ru (A.G.G.); dmt@ioc.ac.ru (A.S.D.); 2 M.V. Lomonosov Moscow State University, Leninskie gory, 119991 Moscow, Russia; E-Mail: fomitskaya.p@gmail.com

**Keywords:** fucosylated chondroitin sulfate, oligosaccharides, synthesis, glycosylation, stereoselectivity, remote participation, sulfation

## Abstract

Natural anionic polysaccharides fucosylated chondroitin sulfates (FCS) from sea cucumbers attract great attention nowadays due to their ability to influence various biological processes, such as blood coagulation, thrombosis, angiogenesis, inflammation, bacterial and viral adhesion. To determine pharmacophore fragments in FCS we have started systematic synthesis of oligosaccharides with well-defined structure related to various fragments of these polysaccharides. In this communication, the synthesis of non-sulfated and selectively *O*-sulfated di- and trisaccharides structurally related to branching sites of FCS is described. The target compounds are built up of propyl β-d-glucuronic acid residue bearing at *O*-3 α-l-fucosyl or α-l-fucosyl-(1→3)-α-l-fucosyl substituents. *O*-Sulfation pattern in the fucose units of the synthetic targets was selected according to the known to date holothurian FCS structures. Stereospecific α-glycoside bond formation was achieved using 2-*O*-benzyl-3,4-di-*O*-chloroacetyl-α-l-fucosyl trichloroacetimidate as a donor. Stereochemical outcome of the glycosylation was explained by the remote participation of the chloroacetyl groups with the formation of the stabilized glycosyl cations, which could be attacked by the glycosyl acceptor only from the α-side. The experimental results were in good agreement with the SCF/MP2 calculated energies of such participation. The synthesized oligosaccharides are regarded as model compounds for the determination of a structure-activity relationship in FCS.

## 1. Introduction

Different types of natural anionic polysaccharides attract increasing attention nowadays due to their biological activity of different types that makes possible their use as pharmacological regulators of several diseases related to biological processes, such as blood coagulation, thrombosis, angiogenesis, inflammation, bacterial and viral adhesion and some others. The most famous biopolymer of the discussed type is glycosaminoglycan heparin, which was found to be a leader on anticoagulant and antithrombotic market for decades [1,2]. Besides, this biopolymer was shown to attenuate metastasis and inflammation in a way of inhibition of P- and l-selectins binding to their cellular ligands [3,4].

Due to several side effects of heparin treatment, new biologically active compounds are intensively searched for and are under development as potential alternative drugs. Among them are fucosylated chondroitin sulfates (FCS) isolated from different sea cucumber species. These polysaccharides demonstrated a wide spectrum of biological activities including anticoagulant, antithrombotic, antitumor, immunostimulatory, anti-hyperglycemia, antiangiogenic, antibacterial, antiviral and some others [5,6,7,8,9,10].

**Figure 1:**
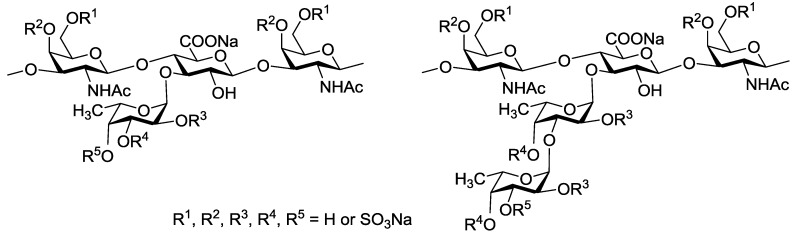
Branched fragments of fucosylated chondroitin sulfates from sea cucumbers.

A number of studied to date FCSs are glycosaminoglycans built up of alternating →4)-linked β-d-glucuronic acid and →3)-linked N-acetyl β-d-galactosamine residues in a backbone. Unlike mammalian chondroitin sulfates, these polysaccharides bear side chains containing *O*-sulfated fucosyl residues attached to *O*-3 of glucuronic acid units (Figure 1) [5,11]. It should be noted that the presence of side chains is essential for biological properties of FCS [12]. The structures of fucosyl-branches vary accordingly to the type of sea cucumber species and determine in many respects the level and the character of its biological activity [5,11]. Moreover, the degree of *O*-sulfation and fucose content in FCS also depend on the geographic range and season of harvesting [13,14]. The known structures of non-sulfated and selectively *O*-sulfated α-l-fucosyl and α-l-fucosyl-(1→3)-α-l-fucosyl fragments which were discovered as the side chains in FCS from sea cucumbers [5,11,15] are shown in Figure 1.

**Figure 2:**
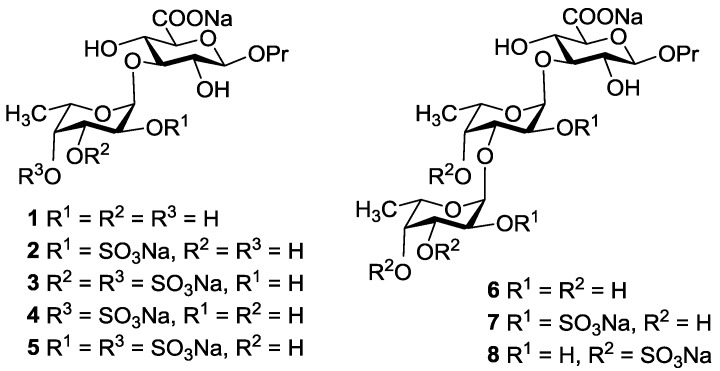
The target compounds **1**–**8** related to branching sites of fucosylated chondroitin sulfates (FCS).

In 2013 Tamura *et al.* reported synthesis of the trisaccharide β-d-GalNAc(4,6-diS)(1→4) [α-l-Fuc(2,4-diS)(1→3)]-β-d-GlcA related to branching site of FCS [16]. But to determine pharmacophore fragments of FCS, a series of compounds with well-defined structure are required. We have started the systematic synthesis of oligosaccharides related to various fragments of these types of natural polysaccharides. In this communication, the first synthesis of non-sulfated and selectively *O*-sulfated di- and trisaccharides **1**–**8** related to the known structures of branching sites of FCS is described (Figure 2). The target compounds are built up of the propyl β-d-glucuronic acid residue bearing at *O*-3 the α-l-fucosyl or α-l-fucosyl-(1→3)-α-l-fucosyl substituents. The sulfation pattern of the fucosyl units was selected according to the data of holothurian FCS structures described in literature (see above for citations).

## 2. Results and Discussion

**Figure 3:**
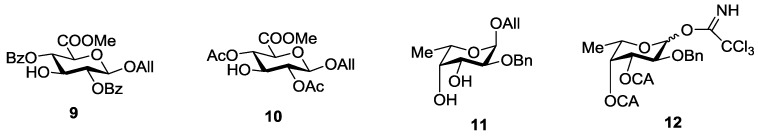
Monosaccharide building blocks applied for the synthesis of FCS related oligosaccharides.

Two monosaccharides **9** and **10** (Figure 3) bearing free hydroxyl group at *C*-3 were studied as glycosyl acceptors for assembling of target compounds **1**–**8**. These compounds were prepared from 2,4-di-*O*-acylated derivatives of 3,6-lactone of allyl glucuronide as described previously [17].

**Figure 4:**
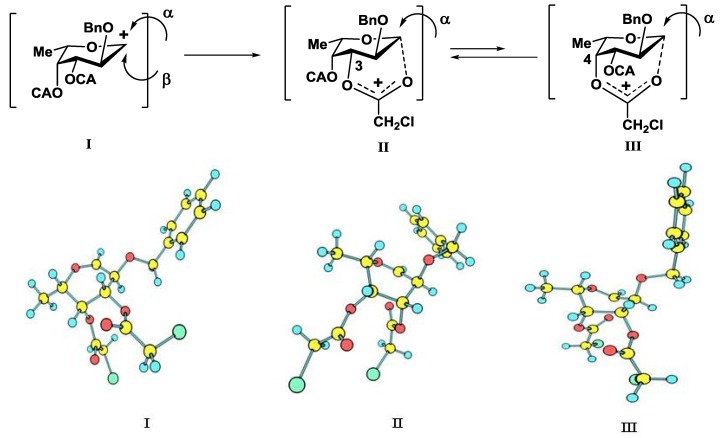
Expected non-stabilized (**I**) and stabilized (**II**), (**III**) fucosyl cations in glycosylation reactions of fucosyl donor **12**. 3D structures of cations were obtained by SCF/MP2 method.

Since the target compounds contain α-l-fucosyl residues, an efficient method for α-l-fucosylation should be applied in their synthesis. Earlier we have shown that the presence of acyl groups at *O*-3 and *O*-4 of the fucosyl donor was essential for α-glycoside formation [18,19,20]. Thus, the use of 2-*O*-benzyl-3-*O*-acetyl-4-*O*-benzoyl-l-fucosyl trichloroacetimidate **13** gave the only α-isomeric glycosylation product [20]. The stereochemical result of the reaction was explained by the remote participation of acyl groups with the formation of the stabilized glycosyl cation (similar to cations **II** and **III** in Figure 4 below), which could be attacked by an acceptor only from the α-side. This approach was quite different then what was used by Tamura *et al.*, where fucosyl fluoride with non-participating allyl and benzyl groups was applied [16].

An additional requirement to the structure of the fucosyl donor is the presence of selectively removable protective groups at *O*-3 and *O*-4, which are necessary for the preparation of the partially *O*-sulfated oligosaccharides **3**–**5** and **8**. It is known that chloroacetyl group (CA) could be selectively removed in the presence of benzoyl and acetyl groups. Thus, we investigated first whether the CA group could stabilize glycosyl cation in the same way as had been observed for other acyls and could thus direct the glycosylation towards the formation of the α-isomer. Following our synthetic strategy, 2-*O*-benzyl-3,4-di-*O*-chloroacetyl trichloroacetimidate **12** was regarded as a donor of choice (Figure 3).

We conducted theoretical calculations to investigate a possibility of the discussed stereocontrolling effect of remote CA groups. At the primary level, as very coarse estimate, we performed molecular mechanics calculations following the technique described in [21] using the evaluation version of HyperChem 1.0 for Linux (HyperChem is the trademark of Hypercube, Inc. (Gainesville, FL, USA) [22]). The MM+ force field (HyperChem version of MM2 [23]) was employed with the electrostatic term using charge-charge interactions. Atomic charges were obtained from single point calculations using semiempirical PM3 approximation [24,25]. Geometry optimizations of a non-stabilized and stabilized forms of the oxocarbenium ion (Figure 4) were conducted and the corresponding “stabilization energies” were calculated as energy differences in pairs E(**I**)—E(**II**) and E(**I**)—E(**III**). At this very low level of theory, it was found that the CA group (especially that at *O*-3) might have the ability to stabilize efficiently the oxocarbenium ion. Stabilization energies were computed as 11.1 and 6.2 kcal/mol for the CA groups at *O*-3 and *O*-4, respectively. Calculated values were only slightly lower than those found for the stabilizing groups in donor **13** (Table 1).

Then the study was carried out at the *ab initio* level with the account for electron correlation. Geometries of all the cations were optimized using the SCF/MP2 approximation with the 6-31+G* basis set as provided along with the NWChem 6.3 software package [26]. This calculation resulted in an increase in the difference in stabilization energies between **12** and **13** (Table 1), but the stabilization energies for the CA groups at *O*-3 and *O*-4 of **12** still remained rather high, suggesting their ability to interact with the cationic center.

**Table 1:** Stabilization energy of the fucosyl cations.

Donor	The Stabilizing Group	Stabilization Energy Calculated at MM+ Level, Kcal/Mole	Stabilization Energy Calculated at SCF/MP2 Level, Kcal/Mole
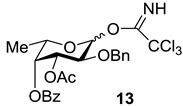	3-*O*-Ac	**11.6**	**15.5**
	4-*O*-Bz	**9.7**	**15.5**
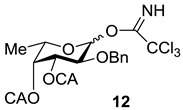	3-*O*-C(O)CH_2_Cl	**11.1**	**10.4**
	4-*O*-C(O)CH_2_Cl	**6.2**	**8.6**

Compound **12** was prepared from diol **11** [27] by per-*O*-chloroacetylation followed by deallylation and subsequent trichloroacetimidation in a yield of 78% over three steps. The efficiency of this donor was then studied in direct experiments.

**Scheme 1:**
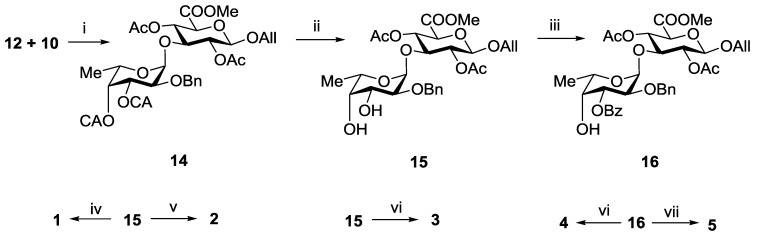
Synthesis of the disaccharides **1**–**5**. Reagents and conditions: (i) TMSOTf, CH_2_Cl_2_, −30 °C, 92%; (ii) (NH_2_)_2_CS, collidine, MeOH, 94%; (iii) Bu_2_SnO, BzCl (1.1 eq), 80%; (iv) (**a**) H_2_, Pd(OH)_2_/C; (**b**) LiOH, H_2_O, THF; (**c**) NaOH, H_2_O, 79%; (v) (**a**) AcCl, Py; (b) H_2_, Pd(OH)_2_/C; (**c**) SO_3_·Py, DMF; (**d**) Amberlite IR-120 (Na^+^), NaHCO_3_; (**e**) LiOH, H_2_O, THF; (f) NaOH, H_2_O, 64%; (vi) (**a**) SO_3_·Py, DMF; (**b**) Amberlite IR-120 (Na^+^), NaHCO_3_; (**c**) H_2_, Pd(OH)_2_/C; (**d**) LiOH, H_2_O, THF; (**e**) NaOH, H_2_O, 62%; (vii) (**a**) H_2_, Pd(OH)_2_/C; (**b**) SO_3_·Py, DMF; (**c**) Amberlite IR-120 (Na^+^); NaHCO_3_; (**d**) LiOH, H_2_O, THF; (**e**) NaOH, H_2_O, 52%.

An attempt to involve the 2,4-di-*O*-benzoylated glucuronyl acceptor **9** into glycosylation with donor **12** in the presence of TMSOTf failed. The main product of the reaction was the respective *N*-glycosylated trichloroacetamide, while compound **9** was recovered unchanged. On the contrary, the 2,4-di-*O*-acetylated glucuronyl acceptor **10** under the same conditions reacted rapidly with the formation of the desired α-linked disaccharide **14** exclusively in a yield of 92% (Scheme 1). The difference in the reactivity of the acceptors **9** and **10** could be explained by the different steric availability of the hydroxyl group at *C*-3. Bulk benzoyl groups at *O*-2 and *O*-4 in **9** hindered the access of glycosyl donor to hydroxyl group. It should be noted that the stereochemical result of the glycosylation was still in good agreement with the theoretical prediction.

Selective removal of CA-groups in **14** using thiourea in the presence of collidine gave diol **15** in a yield of 94% (Scheme 1). This compound was used as the precursor of all target disaccharides **1**–**5**. Thus, hydrogenolysis of **15** followed by saponification gave the non-sulfated propyl glycoside **1**. Per-*O*-sulfation of **15** with subsequent hydrogenolysis and saponification led to the 3′,4′-di-*O*-sulfated disaccharide **3**. Acetylation of **15** followed by hydrogenolysis, *O*-sulfation and saponification gave the 2′-*O*-sulfated disaccharide **2**.

To prepare compounds **4** and **5**, selective 3′-*O*-benzoylation of diol **15** via a stannylidene intermediate was performed with the formation of disaccharide **16** in a yield of 80%. Further *O*-sulfation of **16** and deprotection of the sulfated derivative gave target disaccharide **4**. Hydrogenolysis of **15** followed by *O*-sulfation and saponification gave the 2′,4′-di-*O*-sulfated disaccharide **5**.

**Scheme 2:**
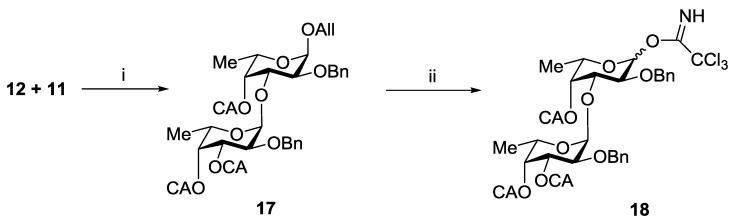
Preparation of the difucoside donor **18**. Reagents and conditions: (i) (**a**) TMSOTf, CH_2_Cl_2_, −30 °C; (**b**) (ClСH_2_CO)_2_O, Py, 78%; (ii) (**a**) PdCl_2_, MeOH; (**b**) CCl_3_CN, Cs_2_CO_3_, 84%.

The synthesis of trisaccharides **6**–**8** was performed using [2 + 1] strategy for the assembling of the carbohydrate chain. Allyl glucuronide **10** was used as an acceptor, and difucoside **18** was chosen as a donor. Compound **18** was prepared by stereo- and regioselective glycosylation of diol **11** with monosaccharide **12** followed by 4-*O*-chloroacetylation, deallylation and trichloacetimidation steps (Scheme 2). Finally, the anomeric mixture of trichloroacetimidates **18** was obtained in a yield of 66%.

**Scheme 3:**
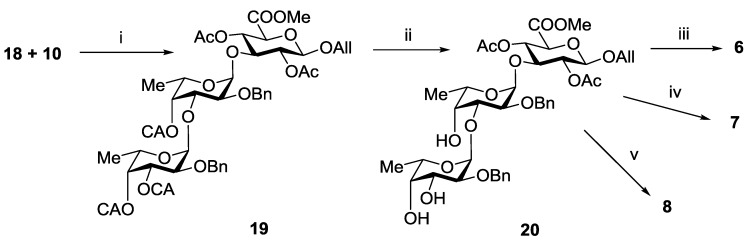
Synthesis of the trisaccharides **6**–**8**. Reagents and conditions: (i) TMSOTf, CH_2_Cl_2_, −30 °C, 90%; (ii) (NH_2_)_2_CS, collidine, MeOH, 92%; (iii) (**a**) H_2_, Pd(OH)_2_/C; (**b**) LiOH, H_2_O, THF; (**c**) NaOH, H_2_O, 82%; (iv) (**a**) AcCl, Py, (**b**) H_2_, Pd(OH)_2_/C; (**c**) SO_3_·Py, DMF; (**d**) Amberlite IR-120 (Na^+^), NaHCO_3_; (**e**) LiOH, H_2_O, THF; (f) NaOH, H_2_O, 54%; (v) (**a**) SO_3_·Py, DMF; (**b**) Amberlite IR-120 (Na^+^), NaHCO_3_; (**c**) H_2_, Pd(OH)_2_/C; (**d**) LiOH, H_2_O, THF; (**e**) NaOH, H_2_O, 62%.

Coupling of thus obtained compounds **18** and **10** in the presence of TMSOTf gave exclusively trisaccharide **19** in a yield of 90% (Scheme 3). Removal of chloroacetyl groups in **19** gave corresponding triol **20**, which was further transformed into target trisaccharides **6**–**8** using the procedure sequences applied for the synthesis of the disaccharides **1**–**3**, respectively.

All the target compounds were characterized with ^1^H and ^13^C NMR spectroscopy (Table 2 and Table 3). The presence of the *O*-sulfate group was confirmed by the downfield shift of the signal of the neighbor proton and carbon atoms in the ^1^H and ^13^C NMR spectra. Introduction of the sulfate group at *O*-2 also influenced the shift of the signals of anomeric proton and carbon atoms.

**Table 2:** ^13^C data for the target compounds **1**–**8**.

Compound	Residue	C1	C2	C3	C4	C5	C6
**1**	→3)-β-d-GlcA	103.2	74.8	83.5	71.7	78.0	176.9
	α-l-Fuc-(1→3	100.7	69.6	70.8	73.2	68.1	16.5
**2**	→3)-β-d-GlcA	103.0	74.7	83.2	71.6	77.9	176.8
	α-l-Fuc-(1→3	98.4	**76.8**	68.7	73.3	68.0	16.5
**3**	→3)-β-d-GlcA	103.5	74.6	83.5	71.6	78.1	176.8
	α-l-Fuc-(1→3	100.5	67.8	**76.6**	**80.4**	67.8	17.0
**4**	→3)-β-d-GlcA	102.1	73.4	82.2	70.5	76.8	176.8
	α-l-Fuc-(1→3	99.1	68.6	68.6	**81.0**	66.4	17.0
**5**	→3)-β-d-GlcA	103.0	74.7	83.2	71.5	77.9	176.8
	α-l-Fuc-(1→3	98.3	**76.6**	67.8	**82.1**	67.4	16.9
**6**	→3)-β-d-GlcA	103.1	74.6	83.6	71.7	78.0	176.8
	→3)-α-l-Fuc-(1→3	100.7	70.7	73.2	69.5	68.1	16.5
	α-l-Fuc-(1→3	96.5	69.3	68.2	75.9	67.9	16.5
**7**	→3)-β-d-GlcA	103.1	75.2	83.2	71.6	78.0	176.9
	→3)-α-l-Fuc-(1→3	98.5	**74.7**	74.3	70.0	68.2	16.5
	α-l-Fuc-(1→3	95.5	**76.5**	68.6	73.4	67.6	16.5
**8**	→3)-β-d-GlcA	102.1	73.4	82.2	70.5	76.8	176.8
	→3)-α-l-Fuc-(1→3	99.1	68.6	74.0	**81.2**	66.4	16.9
	α-l-Fuc-(1→3	100.5	67.8	**76.5**	**80.4**	67.8	17.0

**Table 3:** ^1^H NMR data for the target compounds **1**–**8**.

Compound	Residue	H1	H2	H3	H4	H5	H6
**1**	→3)-β-d-GlcA	4.48	3.51	3.60	3.63	3.74	-
	α-l-Fuc-(1→3	5.27	3.79	3.92	3.82	4.37	1.20
**2**	→3)-β-d-GlcA	4.49	3.55	3.65	3.65	3.74	-
	α-l-Fuc-(1→3	5.57	**4.48**	4.05	3.90	4.44	1.21
**3**	→3)-β-d-GlcA	4.48	3.55	3.67	3.63	3.74	-
	α-l-Fuc-(1→3	5.36	4.00	**4.65**	**4.90**	4.52	1.27
**4**	→3)-β-d-GlcA	4.47	3.50	3.62	3.62	3.73	-
	α-l-Fuc-(1→3	5.30	3.83	4.02	**4.62**	4.50	1.23
**5**	→3)-β-d-GlcA	4.47	3.55	3.63	3.66	3.73	-
	α-l-Fuc-(1→3	5.60	**4.47**	4.17	**4.69**	4.55	1.27
**6**	→3)-β-d-GlcA	4.49	3.55	3.63	3.65	3.74	-
	→3)-α-l-Fuc-(1→3	5.31	3.98	3.84	4.04	4.31	1.21
	α-l-Fuc-(1→3	5.09	3.82	3.95	3.95	4.37	1.21
**7**	→3)-β-d-GlcA	4.49	3.63	3.65	3.67	3.74	-
	→3)-α-l-Fuc-(1→3	5.60	**4.57**	4.07	4.10	4.42	1.24
	α-l-Fuc-(1→3	5.34	**4.45**	4.12	3.91	4.49	1.26
**8**	→3)-β-d-GlcA	4.46	3.55	3.63	3.65	3.73	-
	→3)-α-l-Fuc-(1→3	5.32	4.00	4.08	**4.76**	4.46	1.24
	α-l-Fuc-(1→3	5.20	3.92	**4.62**	**4.90**	4.46	1.27

The synthesized oligosaccharides are regarded as model compounds for the investigation of a structure-activity relationship in FCS. The results of conformational analysis and biological studies of the compounds **1**–**8** will be published elsewhere. Synthesis and interdisciplinary studies of larger oligosaccharides related to structural fragments of FCS are in progress at this laboratory.

## 3. Experimental Section

### 3.1. Materials

Dimethylformamide (DMF, ≥99.5%) and pyridine (≥99.5%) were purchased from Sigma-Aldrich (Saint Louis, MO, USA). Dichloromethane (CH_2_Cl_2_) was successively distilled from diethanolamine, P_2_O_5_, and CaH_2_ under argon. Analytical thin-layer chromatography (TLC) was performed on Silica Gel 60 F_254_ aluminum sheets (Merck, Darmstadt, Germany), and visualization was accomplished using UV light or by charring at ~150 C with 10% (v/v) H_3_PO_4_ in ethanol. Liquid column chromatography was performed on Silica Gel 60, 40–63 µm (Merck, Darmstadt, Germany). Gel chromatography was performed on the Sephadex G-15 column (260 cm) by elution with water at a flow rate of 1 mL/min.

### 3.2. Characterization of Compounds

^1^H and ^13^C NMR spectra were recorded on Bruker DRX500 and AV600 spectrometers at 303 K in CDCl_3_ and D_2_O. Chemical shifts were reported in ppm referenced to the residual CHCl_3_ peak (δ 7.27) for substituted compounds and to acetone peak (0.05% as internal standard, ^1^H δ 2.22 and ^13^C δ 30.9) for the oligosaccharides **1**–**8**. Signal assignment in ^1^H and ^13^C NMR spectra were made using COSY, TOCSY, ROESY and ^1^H-^13^C HSQC techniques. High-resolution mass spectra were acquired by electrospray ionization on a Bruker Daltonics micrOTOF II instrument [28]. Optical rotation values were measured using a JASCO DIP-360 polarimeter at the ambient temperature in solvents specified.

### 3.3. Chemical Synthesis

All glycosylation reactions were carried out under dry argon. Molecular sieves for glycosylation reactions were activated prior to application at 180 °C in vacuum of an oil pump during 2 h.

#### 3.3.1. 2-*O*-Benzyl-3,4-di-*O*-chloroacetyl-l-fucopyranosyl Trichloroacetimidate (**12**)

To a solution of the monosaccharide **11** (800 mg, 2.72 mmol) in CH_2_Cl_2_ (20 mL), Py (2 mL) and (ClCH_2_C(O))_2_O (1 g, 5.9 mmol) were added. The reaction mixture was kept at room temperature (rt) for 1 h, then diluted with EtOAc (50 mL) and washed with HCl (0.1 M) (20 mL) and distilled water (2 × 30 mL). Organic layer was separated and concentrated *in vacuo*. Chromatography of the residue on a silica gel column gave the totally protected monosaccharide as an amorphous solid. The product was dissolved in MeOH (30 mL), and PdCl_2_ (127 mg, 0.8 mmol) was added. The mixture was stirred for 3 h at rt, then it was filtered through a celite pad and the filtrate was concentrated *in vacuo*. Flash column chromatography of the residue on silica gel gave the respective semiacetales as an amorphous solid, which was then dissolved in CH_2_Cl_2_ (15 mL), and CCl_3_CN (300 µL, 3.0 mmol) together with Cs_2_CO_3_ (50 mg, 0.15 mmol) were added. The reaction mixture was stirred for 1 h at rt, then it was filtered through a celite pad and the filtrate was concentrated *in vacuo*. Column chromatography of the residue on a silica gel inactivated by Et_3_N (0.1%) gave the compound **12** as a mixture of α and β isomers in a ratio of 1:1 (1.16 g, 2.12 mmol, 78%). HRMS (ESI) C_19_H_20_Cl_5_NO_7_ [M + Na]^+^ calc.: 571.9580, found: 571.9575.

α-Isomer: *R*_f_ 0.7 (Toluene–EtOAc 3:1); ^1^H NMR (600 MHz, CDCl_3_): δ 1.20 (d, *J*_5,6_ 6.5 Hz, 3H, H-6), 3.99 (d, *J*_gem_ 2.0 Hz, 2H, ClC*H*_2_CO), 4.05 (dd, *J*_1,2_ 3.5 Hz, *J*_2,3_ 10.4 Hz, 1H, H-2), 4.15 (s, 2H, ClC*H*_2_CO), 4.42 (q, 1H, H-5), 4.65 (d, *J*_gem_ 12.0 Hz, 1H, PhC*H*H), 4.74 (d, *J*_gem_ 12.0 Hz, 1H, PhCH*H*), 5.45 (d, *J*_3,4_ 3.1 Hz, 1H, H-4), 5.50 (dd, 1H, H-3), 6.56 (d, *J*_1,2_ 3.5 Hz, 1H, H-1), 7.20–7.40 (m, 5H, Ar), 8.68 (s, 1H, N*H*).

β-Isomer: *R*_f_ 0.6 (Toluene–EtOAc 3:1); ^1^H NMR (600 MHz, CDCl_3_): δ 1.29 (d, *J*_5,6_ 6.5 Hz, 3H, H-6), 3.83 (d, *J*_gem_ 11.7 Hz, 1H, ClCH*H*CO), 3.90 (d, *J*_gem_ 11.7 Hz, 1H, ClC*H*HCO), 3.95 (dd, *J*_1,2_ 8.1 Hz, *J*_2,3_ 10.0 Hz, 1H, H-2), 4.03 (q, 1H, H-5), 4.19 (s, 2H, ClC*H*_2_CO), 4.68 (d, *J*_gem_ 11.4 Hz, 1H, PhC*H*H), 4.92 (d, *J*_gem_ 11.4 Hz, 1H, PhCH*H*), 5.18 (dd, 1H, *J*_3,4_ 3.4 Hz, H-3), 5.34 (d, 1H, H-4), 5.86 (d, *J*_1,2_ 8.1 Hz, 1H, H-1), 7.20–7.40 (m, 5H, Ar), 8.78 (s, 1H, N*H*).

#### 3.3.2. Allyl 2-*O*-benzyl-3,4-di-*O*-chloroacetyl-α-l-fucopyranosyl-(1→3)-(methyl 2,4-di-*O*-acetyl-β-d-glucopyranosyl Uronate) (**14**)

A solution of the monosaccharide **12** (274 mg, 0.5 mmol) and the monosaccharide **10** (166 mg, 0.5 mmol) in CH_2_Cl_2_ (5 mL) was stirred at rt under argon atmosphere with molecular sieves 4 Å (500 mg) for 1 h. The mixture was cooled to −30 °С and TMSOTf of (3 µL) was added. The mixture was stirred for 15 min at −30 °С, then Et_3_N (0.05 mL) was added. The mixture was filtered through a celite pad, and the filtrate was concentrated *in vacuo*. Column chromatography of the residue on silica gel gave the disaccharide **14** as an amorphous solid (330 mg, 0.46 mmol, 92%): *R_f_* 0.4 (Toluene–EtOAc 3:1), [α]_D_ = −63° (*c* 1, EtOAc); ^1^H NMR (600 MHz, CDCl_3_): δ 1.08 (d, *J*_5,6_ 6.6 Hz, 3H, H-6), 1.85 (s, 3H, COC*H*_3_), 2.08 (s, 3H, COC*H*_3_), 3.78 (m, 5H, C(O)OC*H*_3_, H-2^Fuc^, H-3^GlcA^), 3.93 (m, 3H, H-5^GlcA^, ClC*H*_2_CO), 4.10 (s, 2H, ClC*H*_2_CO), 4.12 (m, 1H, CH*H*CH=CH_2_), 4.23 (q, 1H, H-5^Fuc^), 4.37 (m, 1H, C*H*HCH=CH_2_), 4.58 (d, *J*_1,2_ 7.7 Hz, 1H, H-1^GlcA^), 4.62 (s, 2H, PhC*H*_2_), 4.93 (d, *J*_1,2_ 3.3 Hz, 1H, H-1^Fuc^), 5.10 (t, *J*_2,3_ 7.5 Hz, 1H, H-2^GlcA^), 5.20 (m, 3H, H-4^GlcA^, CH_2_CH=C*H*_2_), 5.30 (m, 2H, H-3^Fuc^, H-4^Fuc^), 5.85 (m, 1H, CH_2_C*H*=CH_2_), 7.20–7.40 (m, 5H, Ar). HRMS (ESI) C_31_H_38_Cl_2_O_15_ [M + Na]^+^ calc.: 743.1479, found: 743.1480.

#### 3.3.3. Allyl 2-*O*-benzyl-α-l-fucopyranosyl-(1→3)-(methyl 2,4-di-*O*-acetyl-β-d-glucopyranosyl Uronate) (**15**)

To a solution of the disaccharide **14** (300 mg, 0.42 mmol) in MeOH (5 mL) thiourea (96 mg, 1.26 mmol) and collidine (50 µL) were added. The mixture was kept at 40 °С for 2 h, then it was diluted with EtOAc (20 mL) and washed with HCl (0.1 M) (10 mL) and distilled water (2 × 15 mL). Organic layer was separated and concentrated *in vacuo*. Chromatography of the residue on a silica gel column gave the diol **15** as an amorphous solid (222 mg, 0.39 mmol, 94%): *R_f_* 0.2 (Toluene–EtOAc 1:1), [α]_D_ = −87° (*c* 1, EtOAc); ^1^H NMR (600 MHz, CDCl_3_): δ 1.09 (d, *J*_5,6_ 6.6 Hz, 3H, H-6), 1.82 (s, 3H, COC*H*_3_), 1.98 (s, 3H, COC*H*_3_), 3.23 (s, 2H, 2OH), 3.54 (dd, *J*_1,2_ 3.5 Hz, *J*_2,3_ 10.0 Hz, 1H, H-2^Fuc^), 3.60 (d, *J*_3,4_ 3.3 Hz, 1H, H-4^Fuc^), 3.66 (m, 4H, C(O)OC*H*_3_, H-3^GlcA^), 3.74 (dd, 1H, H-3^Fuc^), 3.83 (d, *J*_4,5_ 9.8 Hz, 1H, H-5^GlcA^), 3.91 (q, 1H, H-5^Fuc^), 4.04 (m, 1H, CH*H*CH=CH_2_), 4.28 (m, 1H, C*H*HCH=CH_2_), 4.49 (d, *J*_1,2_ 7.9 Hz, 1H, H-1^GlcA^), 4.55 (d, *J*_gem_ 11.9 Hz, 1H, PhC*H*H), 4.60 (d, *J*_gem_ 11.9 Hz, 1H, PhCH*H*), 4.77 (d, *J*_1,2_ 3.5 Hz, 1H, H-1^Fuc^), 4.98 (dd, *J*_2,3_ 9.1 Hz, *J*_3,4_ 7.9 Hz, 1H, H-2^GlcA^), 5.03 (t, *J*_3,4_ = *J*_4,5_ 9.3 Hz, 1H, H-4^GlcA^), 5.14 (m, 1H, CH_2_CH=C*H*H), 5.21 (m, 1H, CH_2_CH=CH*H*), 5.76 (m, 1H, CH_2_C*H*=CH_2_), 7.20-7.40 (m, 5H, Ar). HRMS (ESI) C_27_H_36_O_13_ [M + Na]^+^ calc.: 591.2054, found: 591.2050.

#### 3.3.4. Allyl 2-*O*-benzyl-3-*O*-benzoyl-α-l-fucopyranosyl-(1→3)-(methyl 2,4-di-*O*-acetyl-β-d-glucopyranosyl Uronate) (**16**)

A mixture of the compound **15** (210 mg, 0.37 mmol) and Bu_2_SnO (102 mg, 0.41 mmol) in toluene (5 mL) was refluxed with aseotropic removal of water to a volume of 3 mL, treated with benzoyl chloride (50 µL, 0.41 mmol), and the mixture was kept for 1 h. Then MeOH (5 mL) was added, and the mixture was concentrated. Column chromatography of the residue on silica gel afforded the compound **16** (200 mg, 0.30 mmol, 80%): *R_f_* 0.5 (Toluene–EtOAc 1:1), [α]_D_ = −72° (*c* 1, EtOAc); ^1^H NMR (600 MHz, CDCl_3_): δ 1.20 (d, *J*_5,6_ 6.4 Hz, 3H, H-6), 1.86 (s, 3H, COC*H*_3_), 2.08 (s, 3H, COC*H*_3_), 3.78 (s, 3H, C(O)OC*H*_3_), 3.82 (t, *J*_2,3_ 8.6 Hz, *J*_3,4_ 9.0 Hz, 1H, H-3^GlcA^), 3.94 (d, *J*_4,5_ 9.6 Hz, 1H, H-5^GlcA^), 4.02 (m, 2H, H-2^Fuc^, H-4^Fuc^), 4.13 (m, 1H, C*H*HCH=CH_2_), 4.22 (q, 1H, H-5^Fuc^), 4.39 (m, 1H, CH*H*CH=CH_2_), 4.60 (d, *J*_1,2_ 7.6 Hz, 1H, H-1^GlcA^), 4.68 (dd, 2H, PhC*H*_2_), 5.00 (s, 1H, H-1^Fuc^), 5.16 (t, 1H, H-2^GlcA^), 5.22 (m, 2H, H-3^Fuc^, H-4^GlcA^), 5.30 (d, 1H, CH_2_CH=C*H*H), 5.40 (m, 1H, CH_2_CH=CH*H*), 5.88 (m, 1H, CH_2_C*H*=CH_2_), 7.20–8.00 (m, 10H, Ar). HRMS (ESI) C_34_H_40_O_14_ [M + Na]^+^ calc.: 695.2316, found: 695.2321.

#### 3.3.5. Allyl 2-*O*-benzyl-3,4-di-*O*-chloroacetyl-α-l-fucopyranosyl-(1→3)-2-*O*-benzyl-4-*O*-cloroacetyl-α-l-fucopyranoside (**17**)

A solution of the monosaccharide **12** (300 mg, 0.55 mmol) and the monosaccharide **11** (162 mg, 0.55 mmol) in CH_2_Cl_2_ (5 mL) was stirred at rt under argon atmosphere with molecular sieves 4 Å (500 mg) for 1 h. The mixture was cooled to −30 °С and TMSOTf of (5 µL) was added. The mixture was stirred for 15 min −30 °С, then Et_3_N (0.05 mL) was added. The mixture was filtered through a celite pad, and the filtrate was concentrated *in vacuo*. Column chromatography of the residue on a silica gel gave the disaccharide, which was dissolved in CH_2_Cl_2_ (5 mL), and Py (0.5 mL) together with (ClCH_2_C(O))_2_O (150 mg, 0.9 mmol) were added. The reaction mixture was kept at rt for 1 h, then diluted with EtOAc (20 mL) and washed with HCl (0.1 M) (10 mL) and distilled water (2 × 20 mL). Organic layer was separated and concentrated *in vacuo*. Chromatography of the residue on a silica gel column gave the disaccharide **17** as an amorphous solid (326 mg, 0.43 mmol, 78%): *R_f_* 0.6 (Toluene–EtOAc 4:1), [α]_D_ = −170° (*c* 1, EtOAc); ^1^H NMR (600 MHz, CDCl_3_): δ 0.70 (d, *J*_5,6_ 6.4 Hz, 3H, H-6), 1.15 (d, *J*_5,6_ 6.4 Hz, 3H, H-6), 3.80 (m, 2H, H-2^Fuc1^, H-2^Fuc2^), 3.92–4.30 (m, 10H, H-3^Fuc1^, H-5^Fuc1^, 3xClC*H*_2_CO, C*H*_2_CH=CH_2_), 4.50 (q, 1H, H-5^Fuc2^), 4.52–4.80 (m, 4H, 2x PhC*H*_2_), 5.00 (d, 1H, H-1^Fuc1^), 5.10 (d, 1H, H-1^Fuc2^), 5.35 (m, 5H, H-3^Fuc2^, H-4^Fuc1, ^H-4^Fuc2^, CH_2_CH=CH_2_), 5.92 (m, 1H, CH_2_C*H*=CH_2_), 7.20–7.42 (m, 10H, Ar). HRMS (ESI) C_35_H_41_C_l3_O_12_ [M + Na]^+^ calc.: 781.1561, found: 781.1557.

#### 3.3.6. 2-*O*-Benzyl-3,4-di-*O*-chloroacetyl-α-l-fucopyranosyl-(1→3)-2-*O*-benzyl-4-*O*-cloroacetyl-l-fucopyranosyl Trichloroacetimidate (**18**)

To a solution of the disaccharide **17** (230 mg, 0.30 mmol) in MeOH (5 mL) PdCl_2_ (22 mg, 0.12 mmol) was added. The mixture was stirred for 3 h at rt, then it was filtered through a celite pad and the filtrate was concentrated *in vacuo*. Flash column chromatography of the residue on a silica gel gave the respective semiacetales as an amorphous solid, which was then dissolved in CH_2_Cl_2_ (3 mL), and CCl_3_CN (50 µL, 0.49 mmol) together with Cs_2_CO_3_ (10 mg, 0.03 mmol) were added. The reaction mixture was stirred for 1 h at rt, then it was filtered through a celite pad and the filtrate was concentrated *in vacuo*. Column chromatography of the residue on a silica gel inactivated by Et_3_N (0.1%) gave the compound **18** as a mixture of α and β isomers in a ratio of 1:1 (215 mg, 0.25 mmol, 84%). HRMS (ESI) C_34_H_37_Cl_6_NO_12_ [M + Na]^+^ calc.: 884.0345, found: 884.0350.

α-Isomer: *R*_f_ 0.7 (Toluene–EtOAc 4:1); ^1^H NMR (600 MHz, CDCl_3_): δ 0.62 (d, *J*_5,6_ 6.5 Hz, 3H, H-6^Fuc2^), 1.2 (d, *J*_5,6_ 6.5 Hz, 3H, H-6^Fuc1^), 3.80 (dd, *J*_1,2_ 3.4 Hz, *J*_2,3_ 10.5 Hz, 1H, H-2^Fuc2^), 3.84 (d, *J*_gem_ 15.0 Hz, 1H, ClCH*H*CO), 3.90 (d, *J*_gem_ 15.0 Hz, 1H, ClC*H*HCO), 3.94 (d, *J*_gem_ 15.0 Hz, 1H, ClCH*H*CO), 4.04 (m, 4H, H-2^Fuc1^, ClC*H*_2_CO, ClCH*H*CO), 4.33 (m, H-3, H-3^Fuc1^, H-5^Fuc1^, H-5^Fuc2^), 4.50 (d, *J*_gem_ 12.2 Hz, 1H, PhC*H*H), 4.58 (d, *J*_gem_ 10.1 Hz, 1H, PhC*H*H), 4.72 (d, *J*_gem_ 12.2 Hz, 1H, PhCH*H*), 4.80 (d, *J*_gem_ 10.1 Hz, 1H, PhCH*H*), 4.90 (d, *J*_3,4_ 2.7 Hz, 1H, H-4^Fuc2^), 5.10 (d, *J*_1,2_ 3.4 Hz, 1H, H-1^Fuc2^), 5.39 (dd, 1H, H-3^Fuc2^), 5.49 (d, 1H, H-4^Fuc1^), 6.68 (d, *J*_1,2_ 3.4 Hz, 1H, H-1^Fuc1^), 7.20–7.40 (m, 10H, Ar), 8.62 (s, 1H, N*H*).

β-Isomer: *R*_f_ 0.3 (Toluene–EtOAc 4:1); ^1^H NMR (600 MHz, CDCl_3_): δ 0.60 (d, *J*_5,6_ 6.5 Hz, 3H, H-6^Fuc2^), 1.29 (d, *J*_5,6_ 6.5 Hz, 3H, H-6^Fuc1^), 3.78 (dd, *J*_1,2_ 3.5 Hz, *J*_2,3_ 9.5 Hz, 1H, H-2^Fuc2^), 3.81 (d, *J*_gem_ 15.0 Hz, 1H, ClCH*H*CO), 3.92 (m, 4H, H-2^Fuc1^, H-3^Fuc1^, H-5^Fuc1^, ClC*H*HCO), 4.01 (m, 3H, ClC*H*_2_CO, ClCH*H*CO), 4.10 (d, *J*_gem_ 15.2 Hz, 1H, ClCH*H*CO), 4.15 (q, 1H, H-5^Fuc2^), 4.50 (d, *J*_gem_ 12.2 Hz, 1H, PhC*H*H), 4.58 (d, *J*_gem_ 10.1 Hz, 1H, PhC*H*H), 4.72 (d, *J*_gem_ 12.2 Hz, 1H, PhCH*H*), 4.82 (d, *J*_3,4_ 2.9 Hz, 1H, H-4^Fuc2^), 5.05 (d, *J*_1,2_ 3.5 Hz, 1H, H-1^Fuc2^), 5.08 (d, *J*_gem_ 10.1 Hz, 1H, PhCH*H*), 5.37 (dd, 1H, H-3^Fuc2^), 5.40 (d, 1H, H-4^Fuc1^), 5.82 (d, *J*_1,2_ 7.8 Hz, 1H, H-1^Fuc1^), 7.20–7.40 (m, 10H, Ar), 8.75 (s, 1H, N*H*).

#### 3.3.7. Allyl 2-*O*-benzyl-3,4-di-*O*-chloroacetyl-α-l-fucopyranosyl-(1→3)-2-*O*-benzyl-4-*O*-chloroacetyl-α-l-fucopyranosyl-(1→3)-(methyl 2,4-di-*O*-acetyl-β-d-glucopyranosyl Uronate) (**19**)

A solution of the disaccharide **18** (200 mg, 0.23 mmol) and the monosaccharide **10** (76 mg, 0.23 mmol) in CH_2_Cl_2_ (2 mL) was stirred at rt under argon atmosphere with molecular sieves 4 Å (200 mg) for 1 h. The mixture was cooled to −30 °С and TMSOTf of (2 µL) was added. The mixture was stirred for 15 min at −30 °С, then Et_3_N (0.05 mL) was added. The mixture was filtered through a celite pad, and the filtrate was concentrated *in vacuo*. Column chromatography of the residue on a silica gel gave the trisaccharide **19** as an amorphous solid (214 mg, 0.20 mmol, 90%): *R_f_* 0.4 (Toluene–EtOAc 4:1), [α]_D_ = −152° (*c* 1, EtOAc); ^1^H NMR (600 MHz, CDCl_3_): δ 1.06 (d, *J*_5,6_ 6.5 Hz, 6H, H-6^Fuc1^, H-6^Fuc2^), 1.76 (s, 3H, COC*H*_3_), 2.06 (s, 3H, COC*H*_3_), 3.76 (m, 4H, C(O)OC*H*_3_, H-2^Fuc1^), 3.83 (m, 2H, H-3^GlcA^, H-2^Fuc2^), 3.94 (m, 3H, H-5^GlcA^, ClC*H*_2_CO), 4.12 (m, 5H, H-3^Fuc1^, H-5^Fuc1^, CH*H*CH=CH_2_, ClC*H*_2_CO), 4.16 (s, 2H, ClC*H*_2_CO), 4.30 (q, 1H, H-5^Fuc2^), 4.38 (m, 1H, C*H*HCH=CH_2_), 4.55 (d, *J*_gem_ 12.1 Hz, 1H, PhC*H*H), 4.58 (d, *J*_1,2_ 7.7 Hz, 1H, H-1^GlcA^), 4.64 (d, *J*_gem_ 11.2 Hz, 1H, PhC*H*H), 4.69 (d, *J*_gem_ 11.2 Hz, 1H, PhCH*H*), 4.73 (d, *J*_gem_ 12.1 Hz, 1H, PhCH*H*), 4.94 (d, *J*_1,2_ 3.5 Hz, 1H, H-1^Fuc1^), 5.12 (m, 3H, H-2^GlcA^, H-4^GlcA^, H-1^Fuc2^), 5.21 (m, 2H, H-4^Fuc2^, CH_2_CH=CH*H*), 5.30 (m, 1H, CH_2_CH=C*H*H), 5.37 (d, *J*_3,4_ 3.3 Hz, 1H, H-4^Fuc1^), 5.53 (dd, *J*_2,3_ 10.5 Hz, *J*_3,4_ 3.2 Hz, 1H, H-3^Fuc2^), 5.85 (m, 1H, CH_2_C*H*=CH_2_), 7.29–7.44 (m, 10H, Ar). HRMS (ESI) C_46_H_55_Cl_3_O_20_ [M + Na]^+^ calc.: 1055.2250, found: 1055.2246.

#### 3.3.8. Allyl 2-*O*-benzyl-α-l-fucopyranosyl-(1→3)-2-*O*-benzyl-α-l-fucopyranosyl-(1→3)-(methyl 2,4-di-*O*-acetyl-β-d-glucopyranosyl Uronate) (**20**)

To a solution of the trisaccharide **19** (200 mg, 0.19 mmol) in MeOH (3 mL) thiourea (60 mg, 0.78 mmol) and collidine (100 µL) were added. The mixture was kept at 40 °С for 2 h, then it was diluted with EtOAc (20 mL) and washed with HCl (0.1 M) (10 mL) and distilled water (2 × 20 mL). Organic layer was separated and concentrated *in vacuo*. Chromatography of the residue on a silica gel column gave the triol **20** as an amorphous solid (140 mg, 0.17 mmol, 92%): *R*_f_ 0.1 (TolueneEtOAc, 1:1), [α]_D_ = −168 (*c* 1, EtOAc); ^1^H NMR (600 MHz, CDCl_3_): δ 1.18 (d, *J*_5,6_ 6.5 Hz, 3H, H-6^Fuc2^), 1.21 (d, *J*_5,6_ 6.5 Hz, 3H, H-6^Fuc1^), 1.89 (s, 3H, COC*H*_3_), 2.05 (s, 3H, COC*H*_3_), 3.11 (s, 3H, 3OH), 3.64 (d, *J*_3,4_ 2.2 Hz, 1H, H-4^Fuc2^), 3.73 (m, 4H, H-3^GlcA^, H-2^Fuc1^, H-2^Fuc2^, H-4^Fuc1^), 3.75 (s, 3H, C(O)OC*H*_3_), 3.89 (m, 2H, H-5^GlcA^, H-3^Fuc2^), 3.98 (m, 2H, H-3^Fuc1^, H-5^Fuc1^), 4.13 (m, 2H, H-5^Fuc2^, CH*H*CH=CH_2_), 4.38 (m, 1H, C*H*HCH=CH_2_), 4.58 (m, 3H, H-1^GlcA^, PhC*H*_2_), 4.72 (s, 2H, PhC*H*_2_), 4.86 (d, *J*_1,2_ 3.4 Hz, 1H, H-1^Fuc2^), 4.89 (d, *J*_1,2_ 3.6 Hz, 1H, H-1^Fuc1^), 5.10 (t, *J*_2,3_ = *J*_3,4_ 8.5 Hz, 1H, H-2^GlcA^), 5.14 (t, *J*_3,4_ = *J*_4,5_ 8.5 Hz, 1H, H-4^GlcA^), 5.22 (m, 1H, CH_2_CH=CH*H*), 5.30 (m, 1H, CH_2_CH=C*H*H), 5.87 (m, 1H, CH_2_C*H*=CH_2_), 7.20–7.40 (m, 10H, Ar). HRMS (ESI) C_40_H_52_O_17_ [M + Na]^+^ calc.: 827.3102, found: 827.3105.

#### 3.3.9. Propyl α-l-fucopyranosyl-(1→3)-β-d-glucopyranosyl Uronic Acid Sodium Salt (**1**)

To a solution of the disaccharide **15** (57 mg, 0.10 mmol) in MeOH (1.5 mL) and EtOAc (1.5 mL) Pd(OH)_2_/C (15 mg) was added. The mixture was stirred for 1 h at rt under hydrogen atmosphere, then it was filtered through a celite pad, and the filtrate was concentrated *in vacuo*. Column chromatography of the residue on a silica gel (Toluene–EtOAc) gave an amorphous solid, which was dissolved in THF (1.0 mL), and 0.1N_(aq)_ LiOH (0.5 mL) was added. The mixture was kept for 1 h at rt and then 0.1N_(aq)_ NaOH (0.5 mL) was added, and the solution was kept at 40 °C for 1 h. After the mixture was filtered through the Whatman paper filter, the filtrate was concentrated *in vacuo* to a volume of 1 mL. Column chromatography of the residue on the Sephadex G-15 gel in water gave the disaccharide **1** (32 mg, 0.08 mmol, 79%). [α]_D_ = −44° (*c* 0.5, H_2_O). HRMS (ESI) C_15_H_25_NaO_11_ [M − Na]^−^ calc.: 381.1397, found: 381.1402.

#### 3.3.10. Propyl 2-*O*-sulfonato-α-l-fucopyranosyl-(1→3)-β-d-glucopyranosyl Uronic Acid Disodium Salt (**2**)

To a solution of the disaccharide **15** (57 mg, 0.10 mmol) in CH_2_Cl_2_ (1 mL) Py (0.1 mL) and AcCl (0.1 mL) were added. The mixture was kept for 2 h at rt, then it was diluted with EtOAc (20 mL) and washed with HCl (0.1 M) (10 mL) and distilled water (2 × 15 mL). Organic layer was separated and concentrated *in vacuo*. Chromatography of the residue on a silica gel column (Toluene–EtOAc) gave the totally protected disaccharide, which was dissolved in a 1:1 mixture MeOH–EtOAc (3 mL) and Pd(OH)_2_/C (15 mg) was added. The mixture was stirred for 1 h at rt under hydrogen atmosphere, then it was filtered through a celite pad, and the filtrate was concentrated *in vacuo*. Flash chromatography of the residue on a silica gel column (Toluene–EtOAc) gave an amorphous solid, which was dissolved in DMF (1 mL) and PySO_3_ (80 mg, 0.5 mmol) was added. The reaction mixture was kept for 1 h at rt and then quenched with NaHCO_3 _(200 mg). The resin Amberlite IR-120 (Na^+^) and MeOH (1 mL) were added, and the mixture was stirred for 1 h. Then the resin was filtered off, and the filtrate was concentrated *in vacuo* to a volume of 1 mL. Chromatography of the residue on a silica gel column (CH_2_Cl_2_–MeOH) gave the sulfated disaccharide, which was dissolved in THF (1.0 mL), and 0.1N_(aq)_ LiOH (0.5 mL) was added. The mixture was kept for 1 h at rt and then 0.1N_(aq)_ NaOH (0.5 mL) was added, and the solution was kept at 40 °C for 1 h. After the mixture was filtered through the Whatman paper filter, the filtrate was concentrated *in vacuo* to a volume of 1 mL. Column chromatography of the residue on the Sephadex G-15 gel in water gave the disaccharide **2** (33 mg, 0.065 mmol, 64%). [α]_D_ = −38° (*c* 0.6, H_2_O). HRMS (ESI) C_15_H_24_Na_2_O_14_S [M − Na]^−^ calc.: 483.0784, found: 483.0790.

#### 3.3.11. Propyl 3,4-di-*O*-sulfonato-α-l-fucopyranosyl-(1→3)-β-d-glucopyranosyl Uronic Acid Trisodium Salt (**3**)

To a solution of the disaccharide **15** (57 mg, 0.10 mmol) in DMF (1 mL) PySO_3_ (80 mg, 0.5 mmol) was added. The reaction mixture was kept for 1 h at rt and then quenched with NaHCO_3 _(200 mg). The resin Amberlite IR-120 (Na^+^) and MeOH (1 mL) were added, and the mixture was stirred for 1 h. Then the resin was filtered off, and the filtrate was concentrated *in vacuo* to a volume of 1 mL. Chromatography of the residue on a silica gel column (CH_2_Cl_2_–MeOH) gave the sulfated disaccharide, which was dissolved in THF (1.0 mL) and H_2_O (50 µL), and Pd(OH)_2_/C (15 mg) was added. The mixture was stirred for 30 min at rt under hydrogen atmosphere, then it was filtered through a celite pad. To the filtrate 0.1N_(aq)_ LiOH (0.5 mL) was added. The mixture was kept for 1 h at rt and then 0.1N_(aq)_ NaOH (0.5 mL) was added, and the solution was kept at 40 C for 1 h. Then the mixture was filtered through the Whatman paper filter, and the filtrate was concentrated *in vacuo* to a volume of 1 mL. Column chromatography of the residue on the Sephadex G-15 gel in water gave the disaccharide **3** (37 mg, 0.062 mmol, 62%). [α]_D_ = −30° (*c* 0.7, H_2_O). HRMS (ESI) C_15_H_23_Na_3_O_17_S_2_ [M − Na]^−^ calc.: 585.0172, found: 585.0178.

#### 3.3.12. Propyl 4-*O*-sulfonato-α-l-fucopyranosyl-(1→3)-β-d-glucopyranosyl Uronic Acid Disodium Salt (**4**)

To a solution of the disaccharide **16** (55 mg, 0.08 mmol) in DMF (1 mL) PySO_3_ (65 mg, 0.4 mmol) was added. The reaction mixture was kept for 1 h at rt and then quenched with NaHCO_3_ (200 mg). The resin Amberlite IR-120 (Na^+^) and MeOH (1 mL) were added, and the mixture was stirred for 1 h. Then the resin was filtered off, and the filtrate was concentrated *in vacuo* to a volume of 1 mL. Chromatography of the residue on a silica gel column (CH_2_Cl_2_–MeOH) gave the sulfated disaccharide, which was dissolved in THF (1.0 mL) and H_2_O (50 µL), and Pd(OH)_2_/C (15 mg) was added. The mixture was stirred for 30 min at rt under hydrogen atmosphere, then it was filtered through a celite pad. To the filtrate 0.1N_(aq)_ LiOH (0.5 mL) was added. The mixture was kept for 1 h at rt and then 0.1N_(aq)_ NaOH (0.5 mL) was added, and the solution was kept at 40 C for 1 h. Then the mixture was filtered through the Whatman paper filter, and the filtrate was concentrated *in vacuo* to a volume of 1 mL. Column chromatography of the residue on the Sephadex G-15 gel in water gave the disaccharide **4** (24 mg, 0.048 mmol, 62%). [α]_D_ = −32° (*c* 0.6, H_2_O). HRMS (ESI) C_15_H_24_Na_2_O_14_S [M − Na]^−^ calc.: 483.0784, found: 483.0790.

#### 3.3.13. Propyl 2,4-di-*O*-sulfonato-α-l-fucopyranosyl-(1→3)-β-d-glucopyranosyl Uronic Acid Trisodium Salt (**5**)

To a solution of the disaccharide **16** (47 mg, 0.07 mmol) in a 1:1 mixture MeOH–EtOAc (3 mL) Pd(OH)_2_/C (15 mg) was added. The mixture was stirred for 1 h at rt under hydrogen atmosphere, then it was filtered through a celite pad, and the filtrate was concentrated *in vacuo*. Flash chromatography of the residue on a silica gel column (Toluene–EtOAc) gave an amorphous solid, which was dissolved in DMF (1 mL) and PySO_3_ (110 mg, 0.7 mmol) was added. The reaction mixture was kept for 1 h at rt and then quenched with NaHCO_3_ (200 mg). The resin Amberlite IR-120 (Na^+^) and MeOH (1 mL) were added, and the mixture was stirred for 1 h. Then the resin was filtered off, and the filtrate was concentrated *in vacuo* to a volume of 1 mL. Chromatography of the residue on a silica gel column (CH_2_Cl_2_–MeOH) gave the sulfated disaccharide, which was dissolved in THF (1.0 mL), and 0.1N_(aq)_ LiOH (0.5 mL) was added. The mixture was kept for 1 h at rt and then 0.1N_(aq)_ NaOH (0.5 mL) was added, and the solution was kept at 40 C for 1 h. Then the mixture was filtered through the Whatman paper filter, and the filtrate was concentrated *in vacuo* to a volume of 1 mL. Column chromatography of the residue on the Sephadex G-15 gel in water gave the disaccharide **5** (25 mg, 0.04 mmol, 58%). [α]_D_ = −28° (*c* 0.5, H_2_O). HRMS (ESI) C_15_H_23_Na_3_O_17_S_2_ [M − Na]^−^ calc.: 585.0172, found: 585.0176.

#### 3.3.14. Propyl α-l-fucopyranosyl-(1→3)-α-l-fucopyranosyl-(1→3)-β-d-glucopyranosyl Uronic Acid Sodium Salt (**6**)

Treatment of the trisaccharide **20** (40 mg, 0.05 mmol) as described for the preparation of the compound 1 gave the trisaccharide **6** (19 mg, 0.035 mmol, 69%). [α]_D_ = −68° (*c* 0.5, H_2_O). HRMS (ESI) C_21_H_35_NaO_15_ [M − Na]^−^ calc.: 527.1976, found: 527.1981.

#### 3.3.15. Propyl 2-*O*-sulfonato-α-l-fucopyranosyl-(1→3)-2-*O*-sulfonato-α-l-fucopyranosyl-(1→3)-β-d-glucopyranosyl Uronic Acid Trisodium Salt (**7**)

Treatment of the trisaccharide **20** (40 mg, 0.05 mmol) as described for the preparation of the compound **2** gave the trisaccharide **7** (20 mg, 0.027 mmol, 54%). [α]_D_ = −48° (*c* 0.6, H_2_O). HRMS (ESI) C_21_H_33_Na_3_O_21_S_2_ [M − Na]^−^ calc.: 731.0731, found: 731.0736.

#### 3.3.16. Propyl 3,4-di-*O*-sulfonato-α-l-fucopyranosyl-(1→3)-4-*O*-sulfonato-α-l-fucopyranosyl-(1→3)-β-d-glucopyranosyl Uronic Acid Tetrasodium Salt (**8**)

Treatment of the trisaccharide **20** (45 mg, 0.056 mmol) as described for the preparation of the compound **3** gave the trisaccharide **8** (30 mg, 0.035 mmol, 62%). [α]_D_ = −42° (*c* 0.6, H_2_O). HRMS (ESI) C_21_H_32_Na_4_O_24_S_3_ [M − Na]^−^ calc.: 833.0139, found: 833.0143.

### 3.4. Calculations

Starting structures for geometry optimizations were built as follows: for the forms without stabilization, torsional angles H3-C3-O3-CO were set to −40°, C3-O3-CO-O to 0°; and H4-C4-O4-CO to +40°, C4-O4-CO-O to 0°. For the forms with supposed participation of the carbonyl oxygen at O3, torsion H3-C3-O3-CO were set to 180°, and for the supposed participation from O4, torsion H4-C4-O4-CO were set to 180°, putting the corresponding carbonyl oxygen into the proximity of the cationic center.

MM+ optimizations were carried out as described in the text until the RMS gradient attained 0.01 kcal/mol·Å.

*Ab initio* calculations used the SCF/MP2 approach with frozen core orbitals: 1s were frozen for all carbons and oxygens, 1s, 2s and 2p were frozen for chlorines. Optimizations were carried out until the RMS gradient attained the value of 0.0001. After that, vibrational analysis was performed to check that no negative frequencies were present.

## 4. Conclusions

The efficient synthesis of a series of non-sulfated and selectively *O*-sulfated di- and trisaccharides related to branching sites of fucosylated chondroitin sulfates from holothurias has been performed. The synthesized compounds are built up of the propyl β-d-glucuronic acid residue bearing at *O*-3 α-l-fucosyl or α-l-fucosyl-(1→3)-α-l-fucosyl substituents. The sulfation pattern in the fucosyl units was selected according to the known to date holothurian FCS structures. Stereospecific formation of the α-glycoside bond was achieved using 2-*O*-benzyl-3,4-di-*O*-chloroacetyl-α-l-fucosyl trichloroacetimidate as a donor. Stereochemical outcome of the glycosylation was explained by the possible remote participation of the chloroacetyl groups at *O*-3 and *O*-4 with the intermediate formation of the stabilized glycosyl cations, which are hindered from the β-side, and thus could only be attacked by the glycosyl acceptor from the α-side. The experimental results in glycosylation reactions were in good agreement with the SCF/MP2 calculated energies of such participation. Obtained NMR characteristics of synthesized oligosaccharides can be used as model in further analyses of natural FCSs.

## References

[1] Galanaud J.P., Laroche J.P., Righini M. (2013). The history and historical treatments of deep vein thrombosis. J. Thomb. Haemost..

[2] Petitou M., van Boeckel C.A. (2004). A synthetic antithrombin III binding pentasaccharide is now a drug! What comes next?. Angew. Chem. Int. Ed. Engl..

[3] Stevenson J.L., Varki A., Borsig L. (2007). Heparin attenuates metastasis mainly due to inhibition of P- and L-selectin, but non-anticoagulant heparins can have additional effects. Thromb. Res..

[4] Wang L., Brown J.R., Varki A., Esko J.D. (2002). Heparin’s anti-inflammatory effects require glucosamine 6-*O*-sulfation and are mediated by blockade of L- and P-selectins. J. Clin. Invest..

[5] Pomin V.H. (2014). Holothurian Fucosylated Chondroitin Sulfate. Mar. Drugs.

[6] Borsig L., Wang L., Cavalcante M.C., Cardilo-Reis L., Ferreira P.L., Mourão P.A., Esko J.D., Pavão M.S. (2007). Selectin blocking activity of a fucosylated chondroitin sulfate glycosaminoglycan from sea cucumber. Effect on tumor metastasis and neutrophil recruitment. J. Biol. Chem..

[7] Fonseca R.J., Oliveira S.N., Pomin V.H., Mecawi A.S., Araujo I.G., Mourão P.A. (2010). Effects of oversulfated and fucosylated chondroitin sulfates on coagulation. Challenges for the study of anticoagulant polysaccharides. Thromb. Haemost..

[8] Zhao Y., Zhang D., Wang S., Tao L., Wang A., Chen W., Zhu Z., Zheng S., Gao X., Lu Y. (2013). Holothurian glycosaminoglycan inhibits metastasis and thrombosis via targeting of nuclear factor-κB/tissue factor/Factor Xa pathway in melanoma B16F10 cells. PLoS One.

[9] Panagos C.G., Thomson D.S., Moss C., Hughes A.D., Kelly M.S., Liu Y., Chai W., Venkatasamy R., Spina D., Page C.P. (2014). Fucosylated chondroitin sulfates from the body wall of the sea cucumber *Holothuria. forskali*: Conformation, selectin binding, and biological activity. J. Biol. Chem..

[10] Luo L., Wu M., Xu L., Lian W., Xiang J., Lu F., Gao N., Xiao C., Wang S., Zhao J. (2013). Comparison of physicochemical characteristics and anticoagulant activities of polysaccharides from three sea cucumbers. Mar. Drugs.

[11] Myron P., Siddiquee S., Al Azad S. (2014). Fucosylated chondroitin sulfate diversity in sea cucumbers: A review. Carbohydr. Polym..

[12] Mourão P.A., Giumarães B., Mulloy B., Thomas S., Gray E. (1998). Antithrombotic activity of a fucosylated chondroitin sulphate from echinoderm: Sulphated fucose branches on the polysaccharide account for its antithrombotic action. Br. J. Haematol..

[13] Zhao J., Kang H., Wu M., Zeng W., Li Z., Gao Y., Cui J., Wang J., Feng H., Yu L. (2012). Depolymerized Glycosaminoglycan from *Thelenota. ananas* and Preparation Method Thereof. US Patent.

[14] Chen S., Xue C., Yin L., Tang Q., Yu G., Chai W. (2011). Comparison of structures and anticoagulant activities of fucosylated chondroitin sulfates from different sea cucumbers. Carbohydr. Polym..

[15] Kariya Y., Watabe S., Kyogashima M., Ishihara M., Ishii T. (1997). Structure of fucose branches in the glycosaminoglycan from the body wall of the sea cucumber *Stichopus. japonicus*. Carbohydr. Res..

[16] Tamura J., Tanaka H., Nakamura A., Takeda N. (2013). Synthesis of β-d-GalNAc(4,6-diS)(1→4) [α-l-Fuc(2,4-diS)(1→3)]-β-d-GlcA, a novel trisaccharide unit of chondroitin sulfate with a fucose branch. Tetrahedr. Lett..

[17] Kornilov A.V., Sukhova E.V., Nifantiev N.E. (2001). Preparative route to glucuronyl donors bearing temporary protecting group at *O*-3 via 6,3-lactonisation by Bz_2_O or Piv_2_O. Carbohydr. Res..

[18] Gerbst A.G., Ustuzhanina N.E., Grachev A.A., Tsvetkov D.E., Khatuntseva E.A., Whitefield D.M., Berces A., Nifantiev N.E. (2001). Synthesis, NMR and conformational studies of fucoidan fragments. Part 3. Effect of benzoyl group at *O*-3 on stereoselectivity of glycosylation by 3-*O*- and 3,4-di-*O*-benzoylated 2-*O*-benzylfucosyl bromides. J. Carbohydr. Chem..

[19] Komarova B.S., Ustyuzhanina N.E., Tsvetkov Y.E., Nifantiev N.E., Vidal S., Wertz D. (2013). Stereocontrol of 1,2-*cis*-Glycosylation by remote *O*-Acyl protecting groups. Modern Synthetic Methods in Carbohydrate Chemistry—From Monosaccharides to Complex Glycoconjugates.

[20] Ustyuzhanina N., Krylov V., Grachev A., Gerbst A., Nifantiev N. (2006). Synthesis, NMR and conformational studies of fucoidan fragments. part 8: Convergent block-wise synthesis of long chain linear and 2,3-branched oligosaccharides. Synthesis.

[21] Gerbst A.G., Ustuzhanina N.E., Grachev A.A., Tsvetkov D.E., Khatuntseva E.A., Nifant’ev N.E. (1999). Effect of the nature of protecting group at *O*-4 on stereoselectivity of glycosylation by 4-*O*-substituted 2,3-di-*O*-benzylfucosyl bromides. Mend. Commun..

[22] Hypercube, Inc.. http://www.hyper.com.

[23] Allinger N.L. (1977). Conformational analysis. 130. MM2. A hydrocarbon force field utilizing V1 and V2 torsional terms. J. Am. Chem. Soc..

[24] Stewart J.J.P. (1989). Optimization of parameters for semiempirical methods I. Method. J. Comp. Chem..

[25] Stewart J.J.P. (1989). Optimization of parameters for semiempirical methods II. Applications. J. Comp. Chem..

[26] Valiev M., Bylaska E.J., Govind N., Kowalski K., Straatsma T.P., van Dam H.J.J., Wang D., Nieplocha J., Apra E., Windus T.L. (2010). NWChem: A comprehensive and scalable open-source solution for large scale molecular simulations. Comput. Phys. Commun..

[27] Khatuntseva E.A., Ustuzhanina N.E., Zatonskii G.V., Shashkov A.S., Usov A.I., Nifant’ev N.E. (2000). Synthesis, NMR and conformational studies of fucoidan fragments. Part 1: Desulfated 2,3- and 3,4-branched trisaccharide fragments and constituting disaccharides. J. Carbohydr. Chem..

[28] Belyakov P.A., Kadentsev V.I., Chizhov A.O., Kolotyrkina N.G., Shashkov A.S., Ananikov V.P. (2010). Mechanistic insight into organic and catalytic reactions by joint studies using mass spectrometry and NMR spectroscopy. Mendel. Commun..

